# Degradation Potential of the Nonylphenol Monooxygenase of *Sphingomonas* sp. NP5 for Bisphenols and Their Structural Analogs

**DOI:** 10.3390/microorganisms8020284

**Published:** 2020-02-19

**Authors:** Masahiro Takeo, Junichi Akizuki, Aika Kawasaki, Seiji Negoro

**Affiliations:** Department of Applied Chemistry, Graduate School of Engineering, University of Hyogo, 2167 Shosha, Himeji, Hyogo 671-2280, Japan; ym_raskal123-labo@yahoo.co.jp (J.A.); cxj06520@yahoo.co.jp (A.K.); seiji_negoro@yahoo.co.jp (S.N.)

**Keywords:** nonylphenol monooxygenase, bisphenol, diphenylether, *ipso* hydroxylation

## Abstract

The nonylphenol-degrading bacterium *Sphingomonas* sp. strain NP5 has a very unique monooxygenase that can attack a wide range of 4-alkylphenols with a branched side chain. Due to the structural similarity, it can also attack bisphenolic compounds, which are very important materials for the synthesis of plastics and resins, but many of them are known to or suspected to have endocrine disrupting effects to fish and animals. In this study, to clarify the substrate specificity of the enzyme (NmoA) for bisphenolic compounds, degradation tests using the cell suspension of *Pseudomonas putida* harboring the nonylphenol monooxygenase gene (*nmoA*) were conducted. The cell suspension degraded several bisphenols including bisphenol F, bisphenol S, 4,4′-dihydroxybenzophenone, 4,4′-dihydroxydiphenylether, and 4,4′-thiodiphenol, indicating that this monooxygenase has a broad substrate specificity for compounds with a bisphenolic structure.

## 1. Introduction

**Figure 1:**
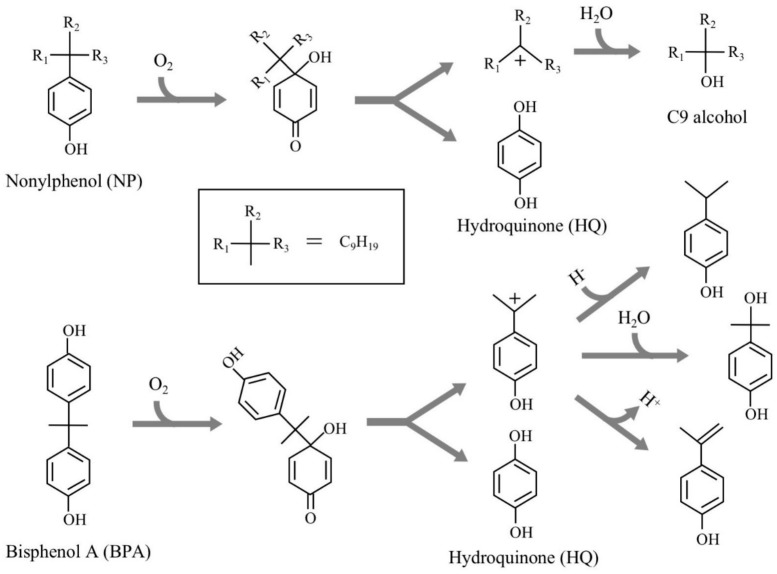
Major degradation pathways of nonylphenol and bisphenol A by *ipso*-hydroxylation (type II *ipso*-substitution) elucidated by Corvini et al. [10], Gabriel et al. [11,12], and Kolvenbach et al. [13].

Various chemical compounds have been synthesized in large quantities to support our comfortable life. However, many of them are known to have the adverse effects on the health of humans and wildlife. One of the emerging adverse effects is the dysfunctions of endocrine (hormonal) systems in animals caused by a part of such chemicals, which are called “endocrine-disrupting chemicals (EDCs)”. Owing to their serious impacts on humans and ecosystem, researchers and research organizations have evaluated many chemicals to identify EDCs using various testing methods over the past three decades. Recently, United Nations Environment summarized many evaluation results and prepared a series of reports including the lists of EDCs and potential EDCs [1,2,3]. Alkylated phenols, bisphenols, and phthalate esters are the representative EDCs. In Japan, 2,2-bis(4-hydroxyphenyl)propane (bisphenol A, BPA), 4-*tert*-octylphenols (OPs), 4-nonylphenols with a branched side chain (NPs), and *o,p*’-dichlorodiphenyltrichloroethane (*o,p*’-DDT) have been recognized as EDCs for fish; this was determined through a series of investigations named SPEED’98, ExTEND2005, ExTEND2010, and ExTEND2016 held by the Ministry of the Environment, Government of Japan [4]. Of them, OPs and NPs have been frequently detected in the environment; they occur in wastewater treatment facilities and ubiquitous aquatic environments as the biodegradation products of nonionic detergents, alkylphenol polyethoxylates, after huge amounts of use and disposal [5,6,7,8]. To understand the biodegradation mechanisms, we isolated *Sphingomonas* sp. strain NP5, an NP-degrading bacterium, from activated sludge after acclimation with NP as the sole carbon source [9]. Two copies of a gene (named *nmoA*) encoding an NP monooxygenase, which catalyzes the initial oxidation of NP, were cloned from strain NP5, and *nmoA* was introduced into *Pseudomonas putida* KT2440 to achieve efficient gene expression. The resulting strain harboring *nmoA* efficiently degraded one of the NP isomers, 4-(1-ethyl-1-methylhexyl)phenol, into hydroquinone (HQ) and 3-methyl-3-octanol [9]. This initial NP oxidation is most likely due to the *ipso*-hydroxylation mechanism, as suggested by Corvini et al. [10] and Gabriel et al. [11] (Figure 1). This mechanism can be applied for NP analogs with a 4-substituted phenolic structure, such as BPA [12,13]. 

BPA has also been used in large quantities as a material for the synthesis of polycarbonate plastics and epoxy resins [7,14]. Owing to the large disposal through industrial activities and daily lives, it has been frequently detected in ubiquitous environments and even in human milk and urine [7,14,15,16,17]. In addition to BPA, some BPA analogs such as bisphenol F (BPF) and bisphenol S (BPS) have also contaminated ubiquitous environments, and thus, they have been detected in such environments at levels similar to those of BPA [14,17]. These BPA analogs are known or suspected to be EDCs [17,18,19,20]. Therefore, it is very important to understand the biodegradability of these compounds. Extensive and intensive research on the biodegradation of BPA revealed that BPA can be degraded by two major mechanisms: oxidative skeletal rearrangement of an aliphatic methyl group in the BPA molecule and hydroxylation of the phenolic rings of BPA followed by ring cleavage [14,21]. However, the information on the biodegradation of other bisphenols (BPs) is limited compared to that of BPA.

In this study, to explore the substrate specificity of the gene product of *nmoA* (NmoA) for BPs, we conducted degradation tests for BPs using the cell suspension of *P. putida* KT2440 expressing *nmoA* and the initial degradability of BPs by this enzyme was briefly evaluated. In addition, some major metabolites from BPs were analyzed to confirm the biodegradation.

## 2. Materials and Methods

### 2.1. Chemical Compounds Used for Degradation Studies

**Figure 2:**
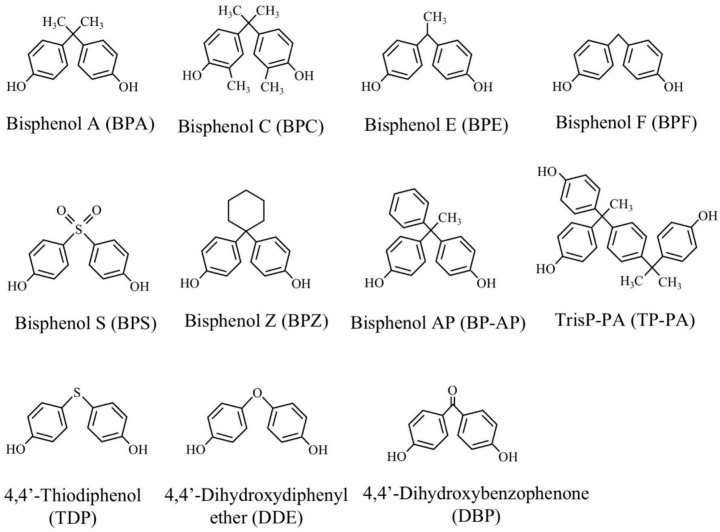
Structures of bisphenols and their structural analogs used.

For the degradation studies, BPA, 2,2-bis(4-hydroxy-3-methylphenyl)propane (bisphenol C, BPC), 1,1-bis(4-hydroxyphenyl)ethane (bisphenol E, BPE), 1,1-bis(4-hydroxyphenyl)cyclohexane (bisphenol Z, BPZ), 4,4′-dihydroxydiphenylether (DDE), 4,4′-dihydroxybenzophenone (DBP), and 4,4′-thiodiphenol (TDP) were obtained from Tokyo Chemical Ind. Co. Ltd. (Tokyo, Japan). Bis(4-hydroxyphenyl)methane (BPF) and 4,4′-dihyroxydiphenylsulfone (BPS) were obtained from Wako Pure Chemical Ind. Ltd. (Osaka, Japan). 1,1-(4-Hydroxyphenyl)-1-phenylethan (bisphenol AP, BP-AP) and α, α-bis(4-hydroxyphenyl)-4-(4-hydroxy-α, α-dimethylbenzyl)-ethylbenzene (TrisP-PA, TP-PA) were kindly gifted from Honshu Chemical Ind. Co. Ltd. (Wakayama, Japan). The chemical structures of these compounds are shown in Figure 2.

### 2.2. Bacterial Strains, Plasmids, Media, and Culture Conditions Used

*P. putida* KT2440 [22] was used as a fast-growing host strain in this study. The strain was revived from the lab freeze stock (in 70% LB medium [23] and 30% glycerol, kept at −80 °C) by incubating aerobically at 30 °C in 10 mL of LB medium for a few days and then kept on LB agar plates at ambient temperature prior to the use. Previously we constructed a recombinant plasmid pBNMOA-F [9], which contained the NP monooxygenase gene (*nmoA*, 1593 bp, accession nos: AB519680 and AB519148) from *Sphingomonas* sp. strain NP5 in the *Bam*HI site of the multicloning site of a broad host range plasmid pBBR1MCS-2 (5.1 kb, with a kanamycin resistance gene) for gram-negative bacteria [24]. Gene expression from the *lac* promoter on the vector seemed to be constitutive in *P. putida* KT2440 [9]. pBNMOA-F was introduced into *P. putida* KT2440 by electroporation as described previously [9]. The recombinant *P. putida* strain was routinely grown in LB medium at 30 °C and at 160 rpm on a rotary shaker. Kanamycin (Km) was added to the medium at 50 mg L^−1^ to maintain the recombinant plasmid in the cell.

### 2.3. Degradation Tests Using Cell Suspension

*P. putida* KT2440 strains with pBNMOA-F (*nmoA*) or pBBR1MCS-2 (vector) were inoculated into LB medium containing Km and incubated overnight. Cells were harvested by centrifugation (7090× *g*, 4 °C, 5 min), washed twice with 10 mM sodium phosphate buffer (pH 7.0), and suspended in the same buffer to an optical density at 600 nm = 4.0. The cell suspension (800 µL each) was distributed into test tubes (14 mm ID × 105 mm), and 10 mM substrate methanol stock solution (16 µL) was added to each test tube to a final concentration of 0.2 mM. After the test tubes of the starting point (0 h) were recovered and stored in a refrigerator, degradation tests were started by shaking the remaining test tubes at 30 °C and at 160 rpm on a rotary shaker. All the test tubes were recovered after shaking for certain intervals (up to 6 h). Then, methanol (800 µL each) was added to each test tube, and the test tubes were vigorously shaken for several minutes to extract the substrate and its metabolites. After the cells and cell debris were removed by centrifugation (21,500× *g*, 4 °C, 10 min), the supernatant was taken and passed through a 0.20 µm membrane filter for further purification. The eluate was subjected to high performance liquid chromatography (HPLC) analysis. Standard substrate samples (0 µM to 250 µM) were also prepared to generate a standard curve through the same processes (once they were introduced into the suspensions of cells with the vector pBBR1MCS-2 and recovered as mentioned above). All the experiments were carried out in triplicate.

### 2.4. HPLC and Gas Chromatography-Mass Spectrometry (GC/MS) Analyses

HPLC analysis was performed using a Shimadzu HPLC system (CBM-20A, 2× LC-10AD, SPD-10A, CTO-10AC, SIL-10A, Shimadzu, Kyoto, Japan) equipped with a Mightysil RP-18GP Aqua column (250 mm × 4.6 mm ID, Kanto Kagaku Kogyo, Tokyo, Japan) under the following conditions: column temp., 40 °C; detection wavelengths, 285 nm and 250 nm; flow rate, 1.0 mL min^−1^; injection volume, 15 µL. A gradient elution was performed using Solvent A (CH_3_OH:H_2_O:CH_3_COOH = 50:950:1) and Solvent B (CH_3_OH:H_2_O:CH_3_COOH = 950:50:1) according to the following program: 100% Solvent A for 5 min; linearly increased to 100% Solvent B for 10 min; 100% Solvent B for 10 min, linearly decreased to the initial condition (100% Solution A) for 5 min. The total analytical time was 30 min. This condition was used for BPA, BPF, and BPS.

As an alternative condition to reduce the analytical time, the following conditions were also employed: a shorter Mightysil RP-18GP Aqua column (150 mm × 4.6 mm ID, Kanto Kagaku Kogyo); column temp., 40 °C; detection wavelengths, 277 nm and 254 nm; flow rate, 1.1 mL min^−1^; injection volume, 10 µL. A gradient elution was performed using Solvent A and Solvent B according to the following program: the initial isocratic mixture of 85% Solvent A and 15% Solvent B for 5 min; linearly increased to 100% Solvent B for 6 min; 100% Solvent B for 2 min; linearly decreased to the initial condition (15% Solution B) for 2 min. The total analytical time was 15 min. This condition was used for BPC, BPE, BPZ, BP-AP, TP-PA, TDP, DDE, and DBP.

For GC/MS analysis of the metabolites from BPs, the abovementioned eluate was transferred to an eggplant flask and evaporated to dryness using a rotary evaporator and a freeze-dryer. The dried matter was dissolved with a small amount of ethylacetate and undissolved matter was removed by centrifugation and membrane treatment as described above. The final elute was analyzed by GC/MS using an Automass 150 system II (JEOL, Tokyo, Japan) equipped with an HP-5 capillary column (J&W Scientific, Folsom, CA, USA) (30 m × 0.32 mm × 0.25 µm) under the following conditions: injection method, split less; sample volume, 1 µL; injection temp., 250 °C; interface temp., 250 °C; source temp., 200 °C; ionization method, EI; ionization potential, 70 eV; ionization current, 300 µA; carrier gas, helium 99.9999%; flow rate, 25 mL min^−1^. The thermal program used was as follows: initial temperature of 60 °C for 2.5 min; increased at 20 °C min^−1^ to 200 °C; 250 °C for 2.5 min. The total analytical time was 14.3 min.

For the analysis of specific metabolites detected in the HPLC analysis, the abovementioned dried matter was dissolved with a small amount of methanol; undissolved matter was removed by centrifugation and membrane treatment as described above. Then, the final eluate was subjected to preparative HPLC using the abovementioned HPLC system with a semipreparative column (Mightysil RP-18GP Aqua, 150 mm × 10 mm ID, Kanto Kagaku Kogyo) and a fraction collector FRC-10A (Shimadzu). The flow rate was set at 3.0 mL min^−1^. The fractionated solution was again dried in the same way. After the dried matter was dissolved with a small amount of ethylacetate, it was subjected to GC/MS analysis described above. 

## 3. Results

### 3.1. Degradation of BPA and BPF

**Figure 3:**
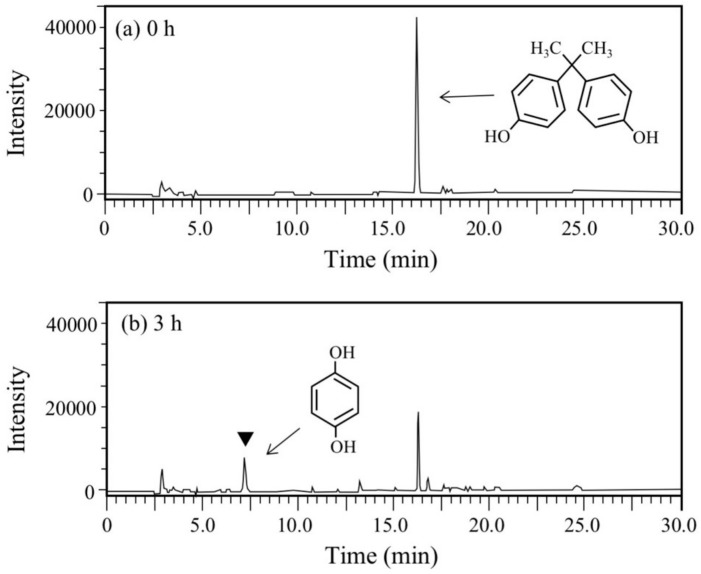
Chromatograms from HPLC analysis of the cell suspension samples of P. putida KT2440 harboring pBNMOA-F (nmoA) in the BPA degradation test. (**a**) Starting point (0 h), and (**b**) after 3-h incubation (3 h). An arrowhead shows a newly appeared peak in the degradation, which was later identified as HQ by GC/MS analysis.

**Figure 4:**
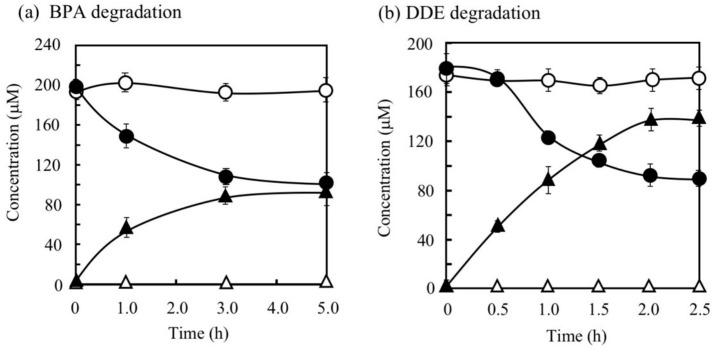
Degradation of bisphenol A (BPA) (**a**) and 4,4′-dihydroxydiphenylether (DDE) (**b**) by *P. putida* KT2440 cells with pBNMOA-F (*nmoA*) or pBBR1MCS-2 (vector) and the corresponding accumulation of HQ. Symbols: circles, BPA or DDE; triangles, HQ; open symbols, cells with vector; closed symbols, cells with *nmoA*. The average ± standard deviation from triplicate tests was shown.

BPA is the most representative compound among BPs as described in the introduction. Thus, in this study, its biodegradability was first evaluated using the cell suspension of *P. putida* KT2440 (*nmoA*). As shown in Figure 3, at the starting point of the degradation test, BPA was detected as a sharp peak at a retention time (rt) of 16.2 min in the HPLC chromatogram, but it decreased with time. After a 3-h incubation, some metabolites were detected as new peaks in the chromatogram (Figure 3), suggesting that BPA was degraded into some metabolites. After a 6-h incubation, the BPA concentration decreased to 46% of the initial concentration (from 200 µM to 92 µM), whereas the BPA concentration in the control cell suspension was almost identical to the initial concentration (Figure 4a). To analyze the major metabolite, the largest peak that appeared at rt of 7.2 min was fractionated by preparative HPLC and analyzed by GC/MS. The mass spectrum had a putative molecular ion of *m/z* = 110 together with two major fragment ion peaks (*m/z* = 81 and 54), which coincided well with the spectrum of authentic HQ (Figure S1). This result demonstrates that HQ was released from BPA by the enzyme activity as proposed in Figure 1. Although some other small peaks were recognized in the chromatogram (Figure 3), we did not analyze other metabolites.

**Figure 5:**
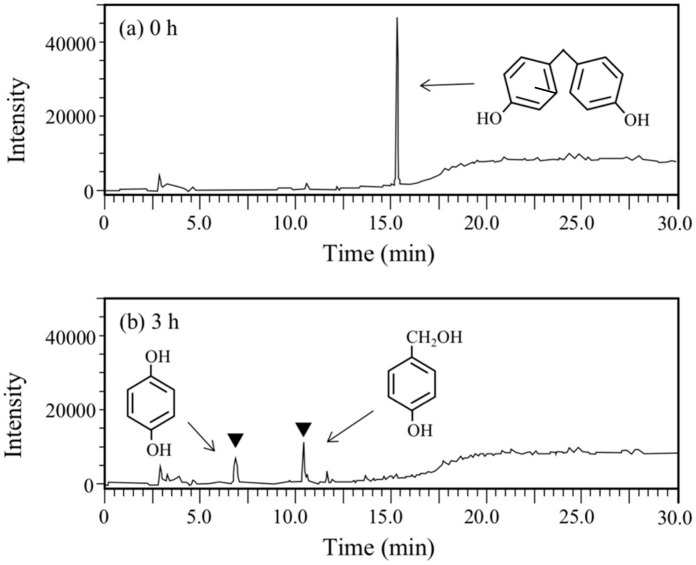
Chromatograms from HPLC analysis of the cell suspension samples of *P. putida* KT2440 harboring pBNMOA-F (*nmoA*) in the BPF degradation test. (**a**) Starting point (0 h), and (**b**) after 3-h incubation (3 h). Arrowheads show newly appeared peaks in the degradation, which were later identified as HQ and 4-hyroxybenzylalcohol by GC/MS and HPLC analyses.

Next, BPF, a simpler analog of BPA, was used for the degradation test. This compound is used as an important substitute for BPA in the manufacturing of plastics and resins [14,17]. At the starting point of the degradation test, a sharp and large peak for BPF was detected at rt of 15.3 min in the HPLC chromatogram (Figure 5a), but it completely disappeared after 6 h (Figure 5b). Instead of the disappearance, two new peaks were observed at rt of 6.8 min and 10.4 min (Figure 5b). The peak with the earlier rt was found to be HQ through the abovementioned GC/MS analysis and another peak was found to be 4-hydroxybenzylalcohol by comparing the rt of the authentic compound and the rt of the spike experiment with the authentic compound. Therefore, BPF was expected to be degraded into HQ and 4-hydroxybenzylalcohol. The latter compound corresponds to one of the BPA metabolites, 2-(4-hydroxyphenyl)isopropanol, in the BPA degradation pathway (Figure 1).

### 3.2. Degradation of Other BPs

In addition to BPA and BPF, degradation tests for BPC, BPE, BPS, BPZ, BP-AP, and TP-PA were also conducted in the same manner. The results are summarized in Table 1, and the HPLC chromatograms of the results are shown in Figure S2 to Figure S5. The decrease in BPC, BPE, BPS, and BPZ by the cell suspension of the recombinant strain with *nmoA* was confirmed by HPLC, whereas no decrease in BP-AP or TP-PA was observed. Therefore, this monooxygenase appears to be unable to attack BPs with three or more aromatic rings. However, it could degrade BPZ with a bulky cyclohexane ring between two phenols. HQ was detected as a metabolite from BPE, BPS, and BPZ (rt = 2.6 min in Figure S2b, rt = 7.2 min in Figure S5b, and rt = 2.7 min in Figure S3a, respectively) (Table 1), suggesting that these BPs were degraded by an *ipso*-hydroxylation mechanism similar to BPA. Except for HQ, two apparent metabolites were detected from BPS and BPZ (Figure S5b and Figure S3a), while three were detected from BPE (Figure S2b). The structure of BPS is different from those of other BPs in that a sulfone structure connects two phenolic rings. Nevertheless, the detection of HQ from BPS revealed that BPS was also degraded into mono-nucleus compounds. In the degradation of BPC, HQ was not detected, because each aromatic ring in BPC had an additional methyl group (instead of HQ, mono-methylated HQ was expected to be the major metabolite from BPC). From BPC, three apparent metabolites were detected (Figure S2a). The degradation ratio of these BPs in 6 h ranged from 45% to 77% (Table 1). This experiment is a 6-h end-point analysis, and thus, the exact comparison of the degradability among BPs is impossible. However, these results demonstrate that the NP monooxygenase NmoA has a broad substrate specificity for simple BPs. 

### 3.3. Degradation of TDP and DBP

TDP is one of the sulfur-containing BPs, in addition to BPS, but it has a sulfide structure linking to two phenols. TDP has higher endocrine disrupting activity relative to BPA [18,25], although less information on its biodegradation is available. TDP can be generated as a metabolite from organophosphorus pesticides such as temphos [25]. In the degradation of TDP, 40% of TDP was degraded in 6 h (Table 1) and only one metabolite was detected (Figure S4b), which was not HQ. 

DBP is composed of two phenols linked by a carbonyl group and can be generated by the biodegradation of BPF and the major UV absorber BP-3 (2-hydroxy-4-methoxybenzophenone) [26,27]. Benzophenones, including DBP, have been demonstrated to have estrogenic activity [28]. In the degradation of DBP, 62% of this compound was degraded in 6 h (Table 1) and some metabolites including HQ (rt = 2.6 min) were detected as tiny peaks in the HPLC chromatogram (Figure S5a). This result indicates that DBP was also degraded into mono-nucleus compounds. Consequently, it was found that NmoA can attack these two compounds. 

### 3.4. Degradation of DDE 

**Figure 6:**
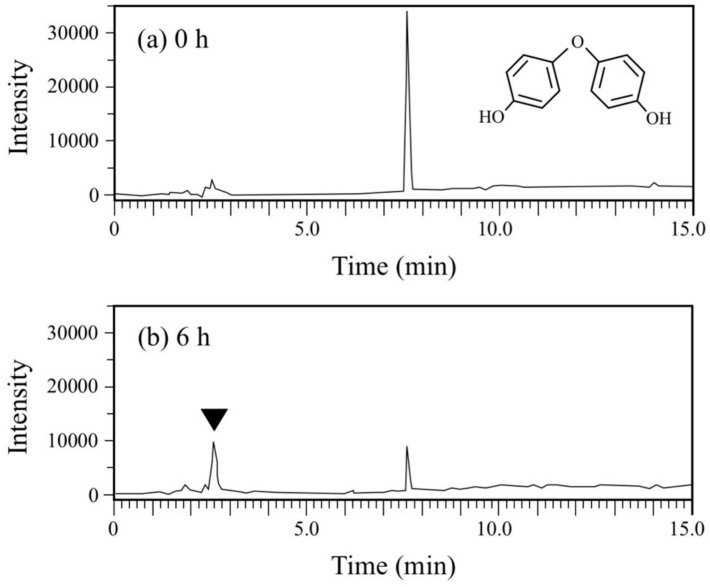
Chromatograms from HPLC analysis of the cell suspension samples of *P. putida* KT2440 harboring pBNMOA-F (*nmoA*) in the BPF degradation test. (**a**) Starting point (0 h), and (**b**) after 6-h incubation (6 h). An arrowhead shows a newly appeared peak in the degradation, which was later identified as HQ by GC/MS analysis.

DDE has an ether bond between two phenols. Generally, diphenylethers with substituent groups have been used as good flame retardants and effective diphenylether herbicides [29,30,31]. However, they have strong adverse effects on humans and other living organisms similar to PCBs and chlorinated dibenzo-p-dioxins [29,30,32,33]. In the DDE degradation, a decrease in the peak for DDE (rt = 7.7 min) was observed in the HPLC chromatogram (Figure 6). In contrast, a peak at rt of 2.6 min corresponding to HQ increased. Finally, 73% of DDE was degraded (from 179 µM to 48 µM) in 6 h. Because the concentration of the HQ produced was much larger than that of the DDE consumed, we repeated the DDE degradation experiment to confirm the stoichiometry in DDE degradation. As shown in Figure 4b, 86 µM of DDE was degraded (from 180 µM to 94 µM) and 138 µM of HQ was produced in 2 h. We extracted the metabolites of DDE from the cell suspension and analyzed them by GC/MS. As a result, in addition to HQ, benzoquinone was also detected as a metabolite (Figure S6). Thus, both of the aromatic rings in DDE might be converted into a major amount of HQ and a small amount of benzoquinone.

## 4. Discussion

Due to the large production and scale of use and the potential endocrine disrupting and toxic effects on living organisms of BPA [7,14,18], the fate of BPA in the environment has been of great concern. There are many studies on BPA biodegradation in the environment, and through such studies, it was found that BPA is susceptible to degradation by bacteria, fungi, algae, and even higher plants under aerobic conditions; however, BPA degradation is poor under anaerobic conditions [34]. As described above, there are two known BPA degradation mechanisms in aerobic bacteria [14,21]. The first mechanism involves the oxidative skeletal rearrangement of an aliphatic methyl group in the BPA molecule, which is caused by two types of oxygenations at the different carbon atoms of BPA: (i) oxygenation at the carbon atoms of the propane moiety (α-quaternary carbon atom or methyl group carbon atom) [35,36,37] and (ii) oxygenation at the carbon atoms of the *ipso*-position of the phenolic rings [12,13,38]. The second mechanism involves (iii) oxygenation at the carbon atoms of the *ortho*-position of the phenolic rings, followed by *meta*-ring cleavage [39].

In the case of (i), after oxygenation, complicated skeletal rearrangements occur at the bridging part between two phenols in BPA to produce mono-nucleus compounds such as 4-hydroxybenzaldehyde, 4-hydroxybenzoic acid, 4-hydroxyacetophenone, and 4-hydroxyphenacylalcohol [36,37]. In the case of (ii), after oxygenation, once an unstable quinone intermediate was formed, it was spontaneously separated into HQ and a carbocation of 4-isopropylphenol [12,13,38]. From the latter, 4-isopropenylphenol, 4-isopropylphenol, and 4-(2-hydroxypropane-2-yl)phenol are nonenzymatically formed (Figure 1). In the case of (iii), as a result of the oxygenation, one of the phenolic rings is converted into a catechol form, followed by the meta-cleavage of the catechol-part [39]. In the cases of (i) and (ii), the 4-hydroxybenzoic acid and HQ formed could serve as a carbon source for the BPA degraders [36,38], while in the case of (iii), the ring-cleavage compounds did not contribute to the growth of the bacterial strain [39]. The BPA reaction by NmoA is categorized into (ii).

Compared to the abovementioned information on the BPA degradation, little is known about the degradation of other BPs. Ike et al. [40] and Danzl et al. [41] evaluated the biodegradability of several BPs including BPA, BPF, and BPS, and showed that under aerobic conditions, the degradability was the following order: BPF, DBP>>BPA>BPE>TDP>BPS. These results show that TDP and BPS with a sulfur atom are more persistent to biodegradation than BPA and that the chemical structure of the bridging part between two phenols can affect the biodegradability.

As a single isolate, some of the BPA-degrading bacteria could also degrade other BPs; the resting cells of *Sphingomonas* sp. MV1 degraded BPC, BPE, BPF, and BPZ [35], while *Sphingomonas* sp. BP-7 also degraded BPC, BPE, and BPZ, but not BPF, BPS, and TDP [42]. These bacterial strains employ (i) for BPA degradation. Therefore, BPC, BPE, and BPZ might be degraded by the same mechanism. However, this degradation mechanism cannot be theoretically applied for BPF, BPS, and TDP, because they do not have the corresponding α-quaternary carbon atom and methyl group as BPA. In fact, the BPF-degrading bacterium *Sphingobium yanoikuyae* FM-2 was isolated from river water, and this bacterium degraded BPF by a different mechanism; once the bridging part of BPF changed to an ester structure by the Baeyer-Villiger reaction, the ester was hydrolyzed into HQ and 4-hydroxybenzoic acid [26]. This bacterium was unable to degrade BPA or other BPs [26]. For BPS and TDP, no assimilating bacteria have been isolated yet, although many BPA-degrading bacteria have thus far been isolated.

The 4-*tert*-butylphenol-degrading bacterium *Sphingobium fuligini*s OMI could degrade BPA by (iii) [39]. This bacterium was able to convert BPF, BPE, DBP, BPS, and TDP into the corresponding catechols. In addition, except for the catechol from BPS, these catechols were further converted into the *meta*-cleavage products. Unfortunately, these products could not be utilized as a carbon source for the growth [39]. Nevertheless, it is worth noting that this bacterium has very unique phenol hydroxylase and *meta*-cleaving dioxygenase with an unexpectedly wide range of substrate specificity for these reactions.

On the other hand, *Sphingomonas* sp. TTNP3 and *Sphingobium xenophagum* Bayram were first isolated as NP-degrading bacteria [43,44], but later, their BPA degradation potential was characterized through the elucidation of their NP degradation mechanisms [12,13]. An OP monooxygenase gene (*opdA*) was cloned from a different OP-degrading bacterium *Sphingomonas* sp. PWE1, and the gene product (OpdA) was found to catalyze the *ipso*-hydroxylation of an OP isomer, 4-(2′,4′,4′-trimethylpentyl)phenol [45]. *opdA* analogs, named *opdA*_TTNP3_ and *opdA*_Bayram_, were found in strains TTNP3 and Bayram, and the gene products were shown to degrade BPA by (ii) [46]. The amino acid sequence identities among these OpdAs are >99.2%, indicating that they are essentially identical [46,47]. In contrast, the amino acid sequence of NmoA showed approximately 83% identity with the OpdAs from strains TTNP3, Bayram, and PWE1. Therefore, the substrate specificity of NmoA may be different from those of the OpdAs. In fact, the substrate specificity of NmoA for NP isomers was considerably different from those of these OpdAs [47], but the difference in the degradability of BPs among these OpdAs and NmoA remains unknown.

This study showed that NmoA has a broad substrate specificity for simple BPs and it could attack BPS and TDP, which are more persistent BPs than BPA [40,41]. The analysis of the metabolites revealed that HQ was one of the metabolites from several BPs. This suggests that these BPs were degraded into mono-nucleus compounds, which may be easily degraded in the environment and by wastewater treatment. *Sphingomonas* sp. NP5 could utilize the HQ formed from NP isomers as a carbon source and grow slowly on NP isomers. However, it could not grow on BPA, although HQ can be generated from BPA by NmoA. The reason is unclear, but there might be some problems in HQ metabolism such as the insufficient induction of HQ-assimilating enzymes by BPA. In contrast, *Cupriavidus basilensis* JF1 was able to grow slowly on BPA, and it is expected to employ (ii) by the analysis of the intermediates from BPA [38]. This bacterium must have a NmoA-like enzyme and an HQ-assimilating system, although the genes and enzymes related to BPA degradation are unknown. However, this suggests that even more persistent BPs can also be assimilated by bacterial strains with an NmoA-like enzyme if they have efficient assimilation systems for mono-nucleus metabolites such as HQ. At present, there are no reports on bacterial strains that can assimilate BPS and TDP, but it is possible to make such a bacterial strain by introducing *nmoA* into a mono-nucleus metabolite-assimilating bacterium.

The concentrations of BPs used in this study were approximately 200 µM (corresponding to 45.6 mg L^-1^ in BPA), which is much higher than the reported concentrations of BPA in surface water (up to 92 µg L^-1^) and in groundwater (up to 20 µg L^−1^) [7]. However, BPs tend to be absorbed to suspended solids in water due to their hydrophobic properties. Therefore, high concentrations of BPs have been detected in sludges and sediments in industrial and metropolitan areas [for example, 13.3 mg kg^−1^ (dry weight) of BPA and 25.3 mg kg^−1^ (dry weight) of total BPs in the sediment of Lake Shihwa, South Korea] [48]. Such accumulation of BPs is likely associated with the discharge of wastewaters from chemical plants around the place [48]. In fact, direct wastewaters from industrial processes contain higher concentrations of BPs (for example, >10 mg L^−1^ of BPA in the pulping process of a waste paper recycling plant) [49]. In addition, a high concentration of BPA (17.2 mg L^−1^) was detected in the leachate of a landfill site [50]. Thus, the constructed recombinant strain harboring *nmoA* would be useful for the treatment of wastes/wastewaters severely contaminated with BPs and bioremediation for the hot spots.

## 5. Conclusions

Bisphenolic comounds are important industrial materials but known to or suspected to be EDCs. To understand the biodegradation potential of the nonylphenol monooxygenase of *Sphingomonas* sp. NP5 for BPs and their analogs, degradation tests for these compounds were conducted using the cell suspension of the recombinant *P. putida* KT2440 harboring the enzyme gene (*nmoA*). As a result, the KT2440 cells showed the degradability for several BPs including BPA, BPE, BPF, and BPS. They also showed the degradability for BP analogs such as TDP, DBP, and DDE. This result indicates that this monooxygenase has a broad substrate specificity for compounds with a bisphenolic structure.

## Figures and Tables

**Table 1:** Degradability of BPs by *P. putida* KT2440 harboring pBNMOA-F (*nmoA*).

Compound	Initial Concentration (µM) ^1^	Final Concentration (µM) ^1^	Degradation Ratio (%) ^2^	Detection of HQ
BPA	200 ± 10	92 ± 6	54	+
BPC	258 ± 8	142 ± 7	45	−
BPE	139 ± 4	46 ± 2	67	+
BPF	139 ± 4	0	100	+
BPS	172 ± 8	40 ± 16	77	+
BPZ	193 ± 8	80 ± 6	59	+
BP-AP	273 ± 18	275 ± 5	0	−
TP-PA	141 ± 9	150 ± 18	0	−
TDP	182 ± 3	110 ± 2	40	−
DBP	143 ± 2	55 ± 2	62	+
DDE	179 ± 8	48 ± 2	73	+

^1^ The average ± standard deviation from triplicate tests was shown. ^2^ (Initial conc. − Final conc.)/Initial conc.

## Supplementary Materials

Supplementary materials can be found at https://www.mdpi.com/2076-2607/8/2/284/s1. Figure S1: GC/MS analysis of the metabolite detected in the degradation of BPA. Figure S2: Chromatograms from HPLC analysis of the cell suspension samples of *P. putida* KT2440 harboring pBNMOA-F (*nmoA*) in the degradation of BPC (a) and the degradation of BPE (b). Figure S3: Chromatograms from HPLC analysis of the cell suspension samples of *P. putida* KT2440 harboring pBNMOA-F (*nmoA*) in the degradation of BPZ (a) and the degradation of BP-AP (b). Figure S4: Chromatograms from HPLC analysis of the cell suspension samples of *P. putida* KT2440 harboring pBNMOA-F (*nmoA*) in the degradation of TP-PA (a) and the degradation of TDP (b). Figure S5: Chromatograms from HPLC analysis of the cell suspension samples of *P. putida* KT2440 harboring pBNMOA-F (*nmoA*) in the degradation of DBP (a) and the degradation of BPS (b). Figure S6: GC/MS analysis of the metabolite detected in the degradation of DDE.
