# Supplementary material for: Degradation Potential of the Nonylphenol Monooxygenase of Sphingomonas sp. NP5 for Bisphenols and Their Structural Analogs
Source: Microorganisms. 2020 Feb 19;8(2):284. doi: 10.3390/microorganisms8020284 (PMC7074866; doi:10.3390/microorganisms8020284)
Supplement: Supplementary file 1 [file microorganisms-08-00284-s001.pdf]

International Journal of
Molecular Sciences

MDPI

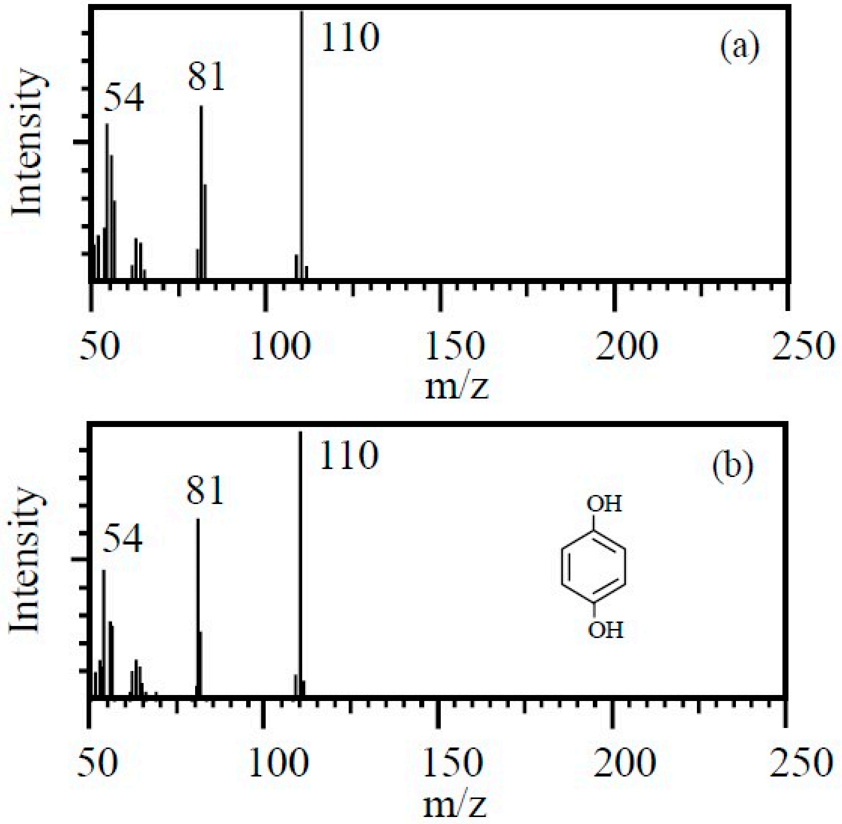

Figure S1. GC/MS analysis of the metabolite detected in the degradation of BPA. (a) Metabolite, (b) authentic hydroquinone.

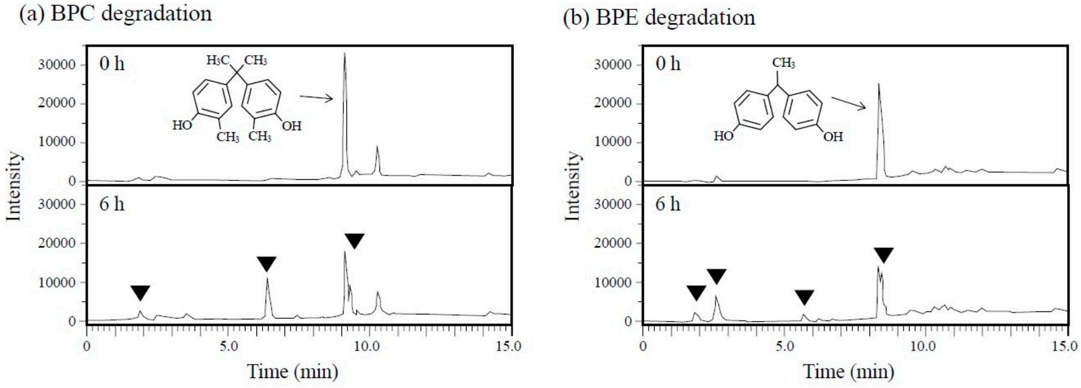

Figure S2. Chromatograms from HPLC analysis of the cell suspension samples of *P. putida* KT2440 harboring pBNMOA-F (*nmoA*) in the degradation of BPC (a) and the degradation of BPE (b). Arrowheads show putative metabolites from BPC or BPE.

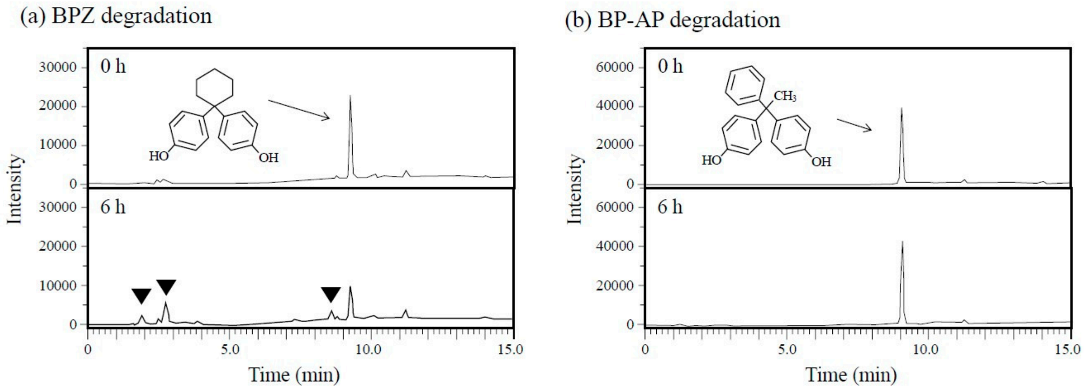

Figure S3. Chromatograms from HPLC analysis of the cell suspension samples of *P. putida* KT2440 harboring pBNMOA-F (*nmoA*) in the degradation of BPZ (a) and the degradation of BP-AP (b). Arrowheads show putative metabolites from BPZ.

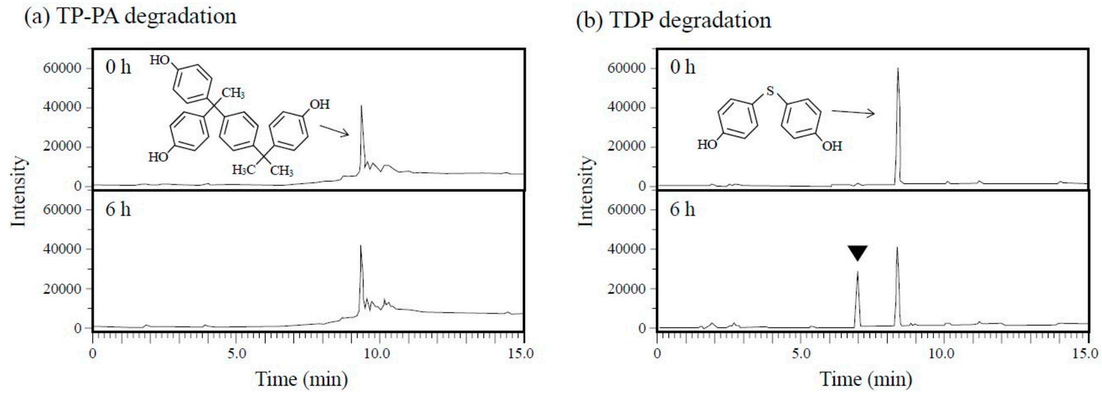

Figure S4. Chromatograms from HPLC analysis of the cell suspension samples of *P. putida* KT2440 harboring pBNMOA-F (*nmoA*) in the degradation of TP-PA (a) and the degradation of TDP (b). An arrowhead shows a putative metabolite from TDP.

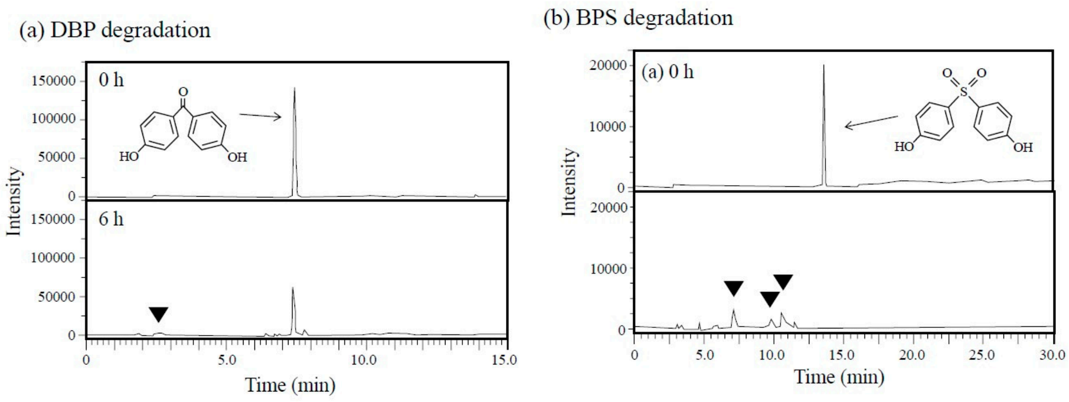

Figure S5. Chromatograms from HPLC analysis of the cell suspension samples of *P. putida* KT2440 harboring pBNMOA-F (*nmoA*) in the degradation of DBP (a) and the degradation of BPS (b). Arrowheads show putative metabolites from DBP or BPS.

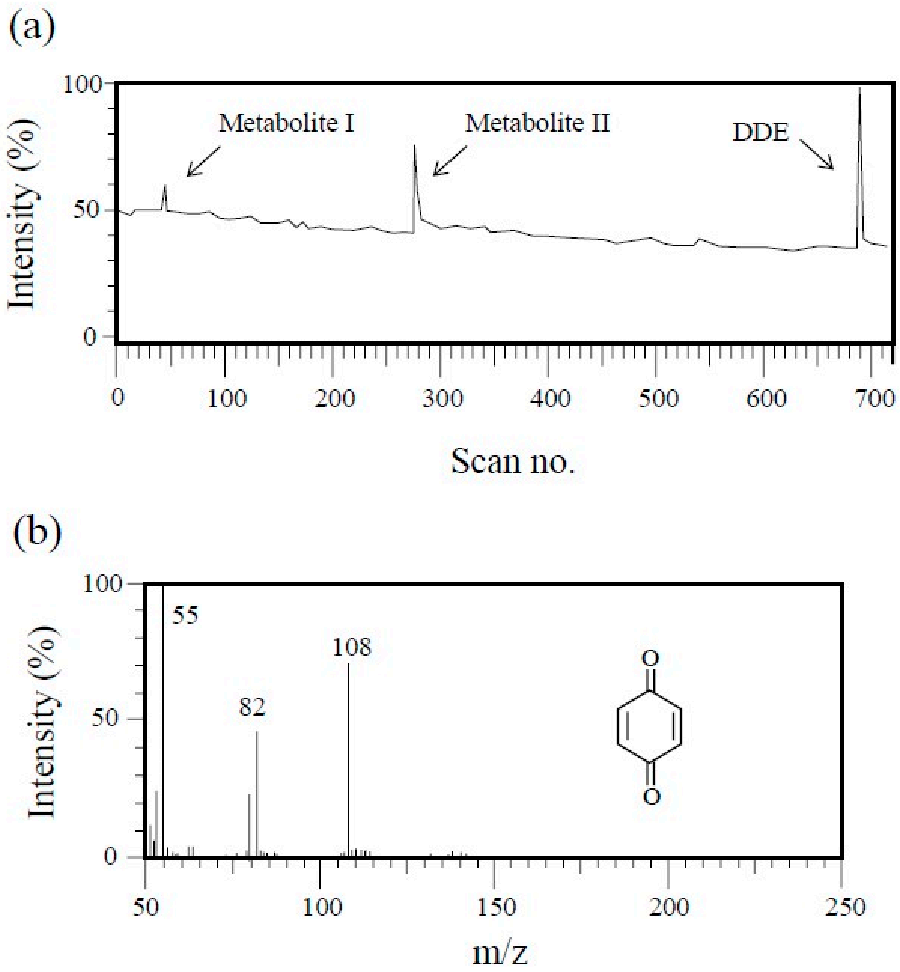

Figure S6. GC/MS analysis of the metabolite detected in the degradation of DDE. (a) Mass chromatogram, (b) mass spectrum of Metabolite I.
